# Supplementary material for: Paraventricular hypothalamus mediates diurnal rhythm of metabolism
Source: Nat Commun. 2020 Jul 30;11:3794. doi: 10.1038/s41467-020-17578-7 (PMC7393104; doi:10.1038/s41467-020-17578-7)
Supplement: Supplementary file 1 — Supplementary Information [file 41467_2020_17578_MOESM1_ESM.pdf]

Kim et al, Paraventricular Hypothalamus Mediates Diurnal Rhythm of Metabolism

Supplementary Figures (Total 9 Figures)

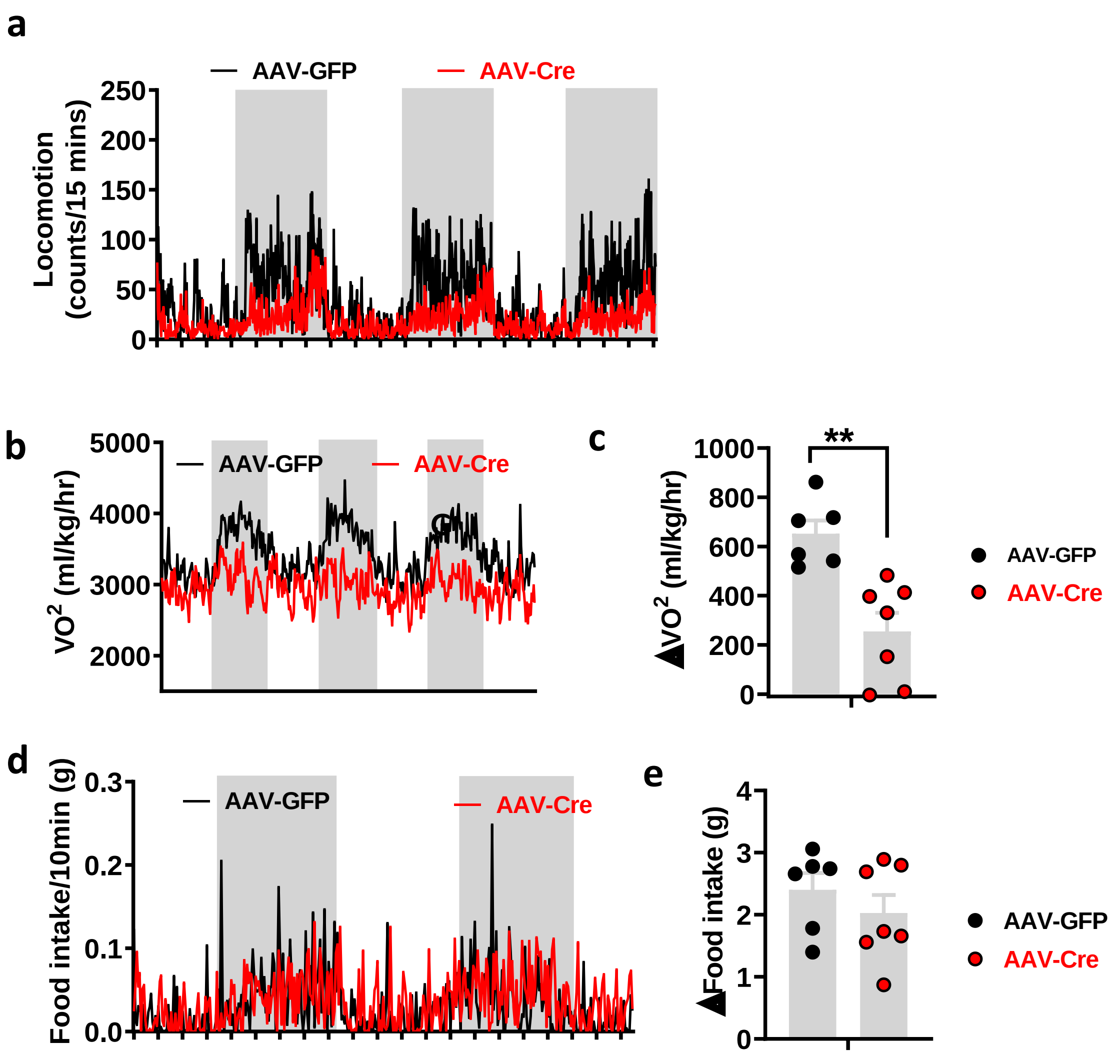

Supplementary Fig. 1. Deletion of BMAL1 in the PVH caused disrupted diurnal patterns in energy expenditure and feeding.

Bmal1$^{flox/flox}$ mice (8-10 weeks old) received bilateral injections of AAV-GFP or AAV-Cre-GFP and were followed for body weight, feeding and expenditure. (a) Locomotor activity measured in CLAMS 3 weeks after viral delivery. Energy expenditure (b-c, n=6-8 males) and feeding (d-e, n=6-8 males) were measured 8-9 weeks after viral delivery. The difference in energy expenditure between day and night periods was highly significant between groups (b, unpaired 2-tailed Student's t test, n=7 each, t=4.127, df=11, **p=0.0017) and there was a trend to a reduced difference in feeding between day and night in AAV-Cre mediated deletion group, but it was not different (e). Data presented at mean +/- SEM.

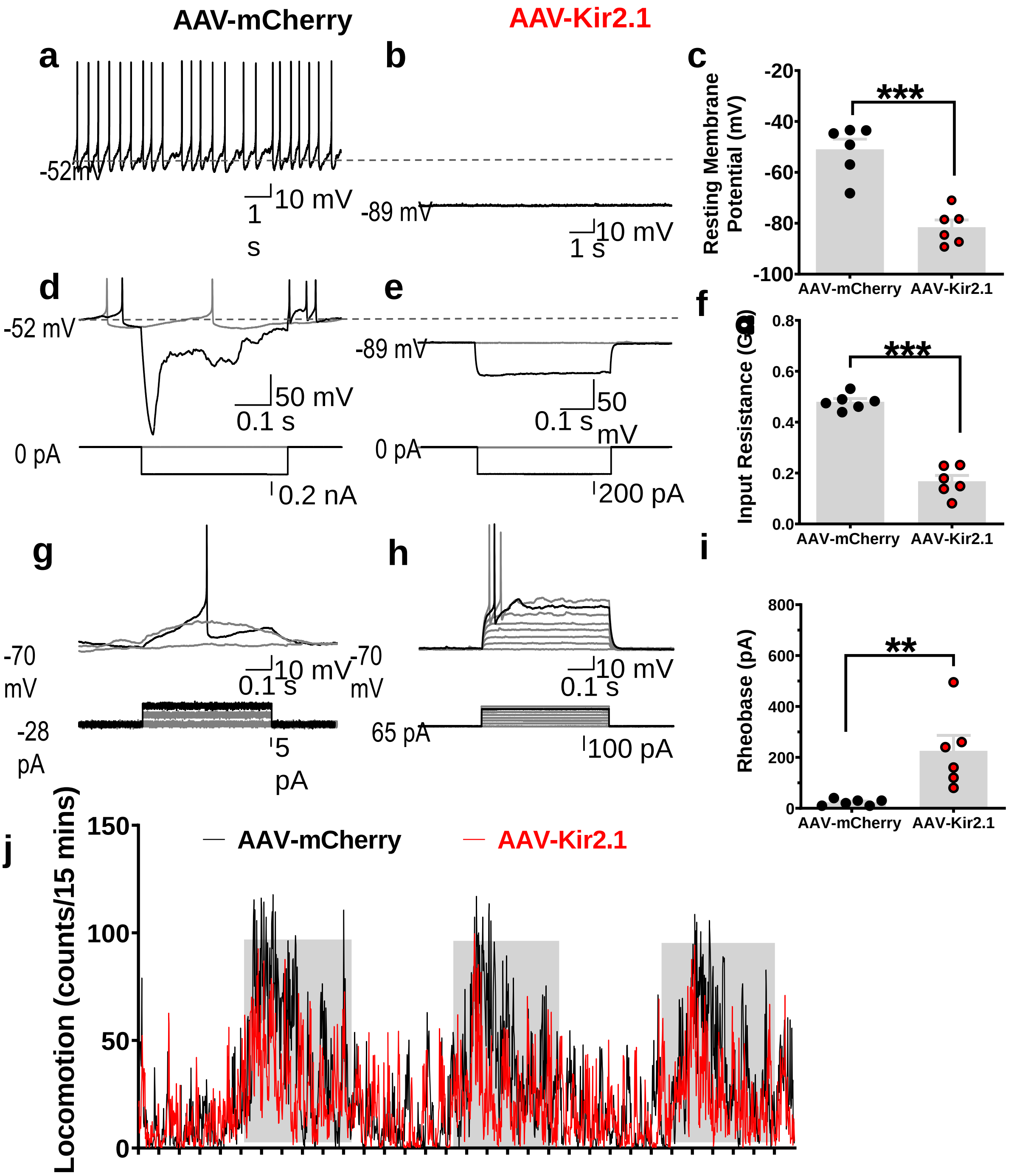

Supplementary Fig. 2. Expression of Kir2.1 led to a reduced activity level of PVH Sim1 neurons.

Sim1-Cre mice (8-10 weeks old) received injections of AAV-FLEX-mCherry or AAV-FLEX-Kir2.1-tdTomato vectors to bilateral PVH and used for studies. (a-c) Representative recording traces showing spontaneous action potential firing in control (a) and no firing in Kir2.1 neurons (b), and comparison of resting membrane potential between groups (c, unpaired 2-tailed Student's t test, n=6 each, t=6.223, df=10, ***p<0.0001). (d-e) Representative traces on voltage changes by injecting currents in control (d) and Kir2.1 neurons (e), and comparison of input resistance between groups (f, unpaired 2-tailed Student's t test, n=6 each, t=6.223, df=10, ***p<0.0001). (g-i) Representative traces on elicitation of action potentials by injecting currents to test minimum current injections (Rheobase) required for action potential firing in control (g) and Kir2.1 neurons (h), and comparison of rheobase between groups (I, unpaired 2-tailed Student's t test, n=6 each, t=3.324, df=10, **p=0.0077). (j) Locomotion measured by CLAMS 2-3 weeks after viral delivery.

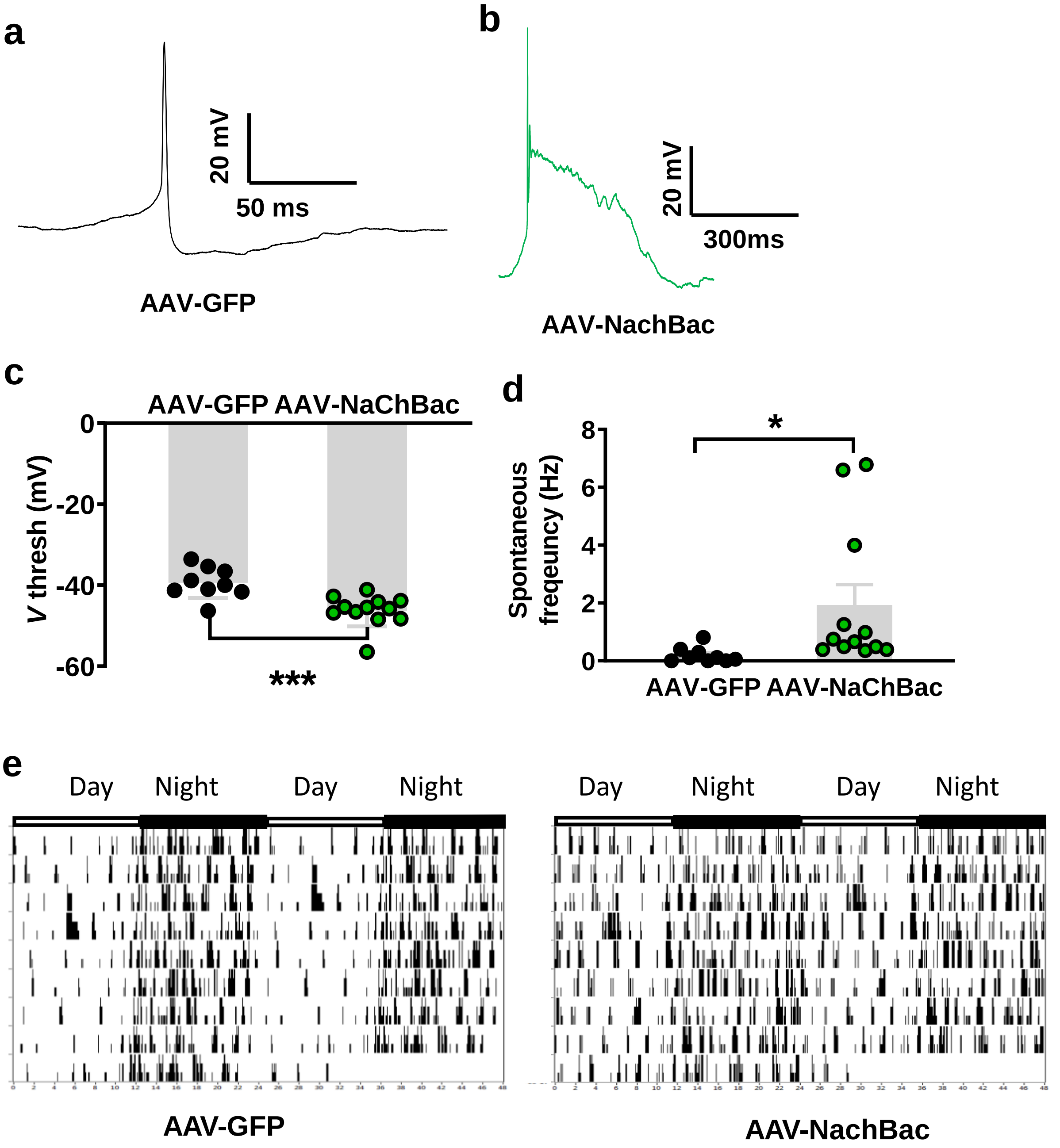

Supplementary Fig. 3. Expression of NachBac led to an increased level of neuron activity.
Sim1-Cre mice (8-10 weeks old) received injections of AAV-FLEX-GFP or AAV-FLEX-NachBac-Venus vectors to bilateral PVH and used for studies. Representative traces showing typical action potential in control (a) and NachBac (b) neurons. Comparison in the threshold for action potential firing (c, unpaired 2-tailed Student's t test, n=9 for GFP or 11 for NachBac, t=4.037, df=19, ***p=0.0007) and frequency of spontaneously firing (d, unpaired 2-tailed Student's t test, n=9 for GFP or 11 for NachBac, t=2.102, df=19, *p=0.0491). (e) Voluntary locomotion in home cage measured by running wheels 7-8 weeks after viral delivery.

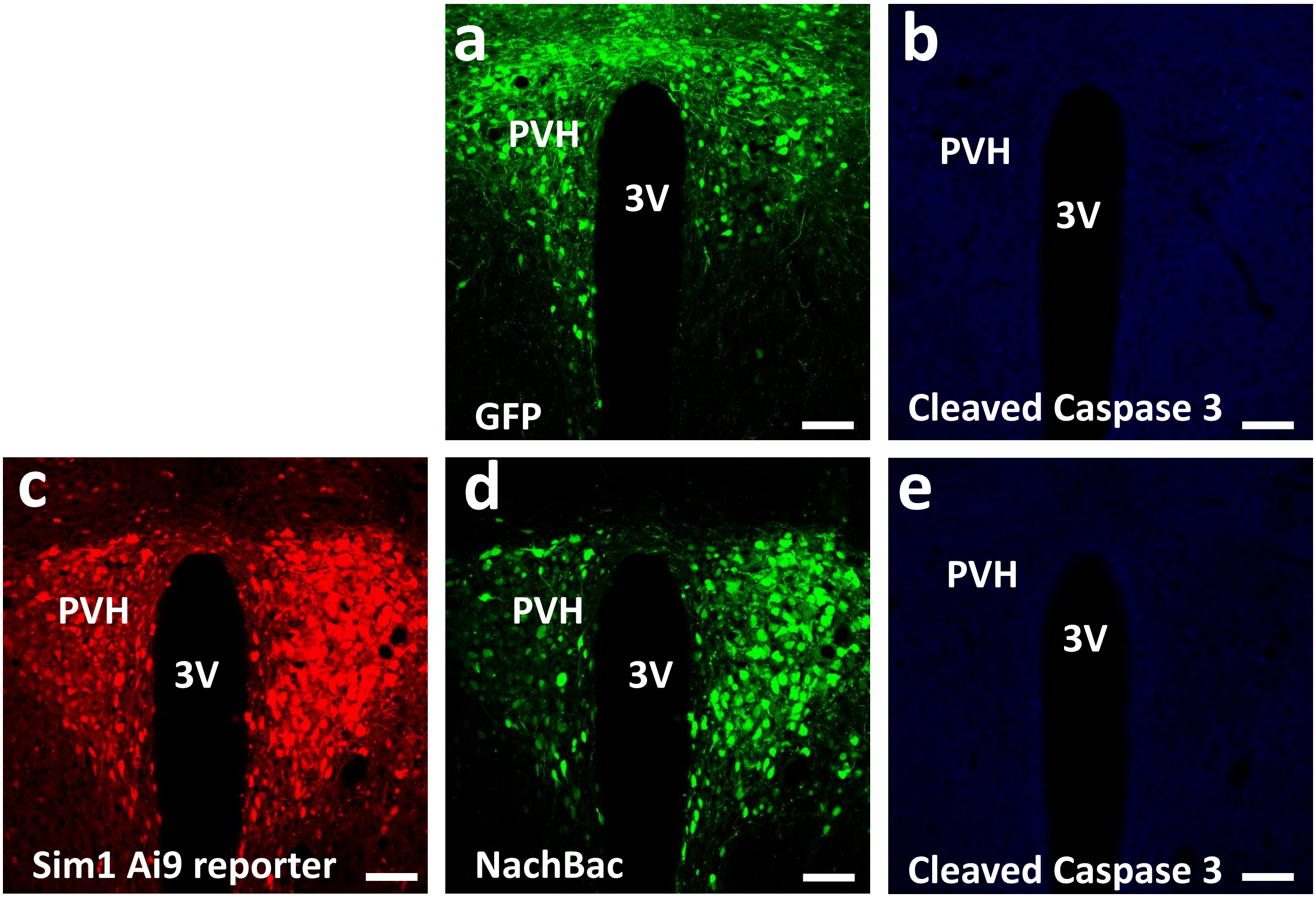

Supplementary Fig. 4. Assessment of apoptosis in neurons with expression of NachBac. Immunostaining was performed on PVH-containing brain sections from controls (a and b) and NachBac mice (c-e). (a-b) Control brain sections showing expression of GFP (a) and cleaved caspase 3 (b). (c-e) Nachbac mouse brain sections showing Sim1 neurons (c, Ai9 reporter), NachBac expression (d) and cleaved caspase 3 (e). No difference was observed in cleaved caspase 3 expression between groups. Similar observations have been observed in additional mice (total n=3 each/group). Scale bars: 50 µm.

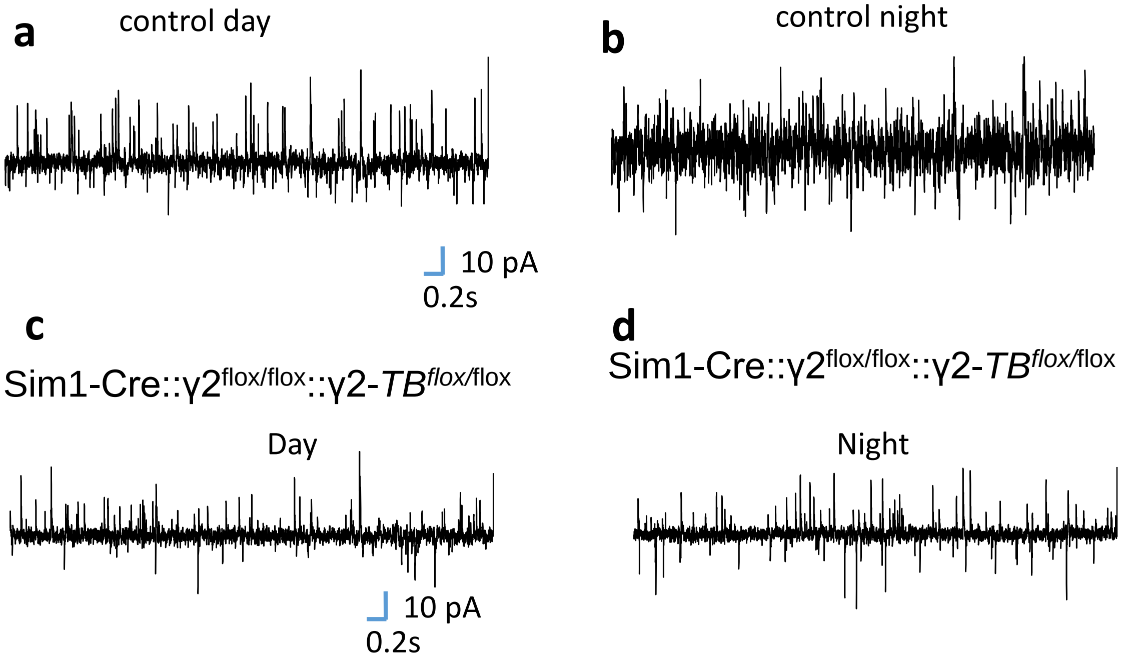

Supplementary Fig. 5. Representative traces showing IPSC (upward currents) and EPCS (downward currents) with recording using voltage clamped at -40 mV in control (a and b) and in Sim1-Cre::γ2$^{flox/flox}$::γ2-*TB*$^{flox/flox}$ mice (C and D) at day (a and c) or night (b and d).

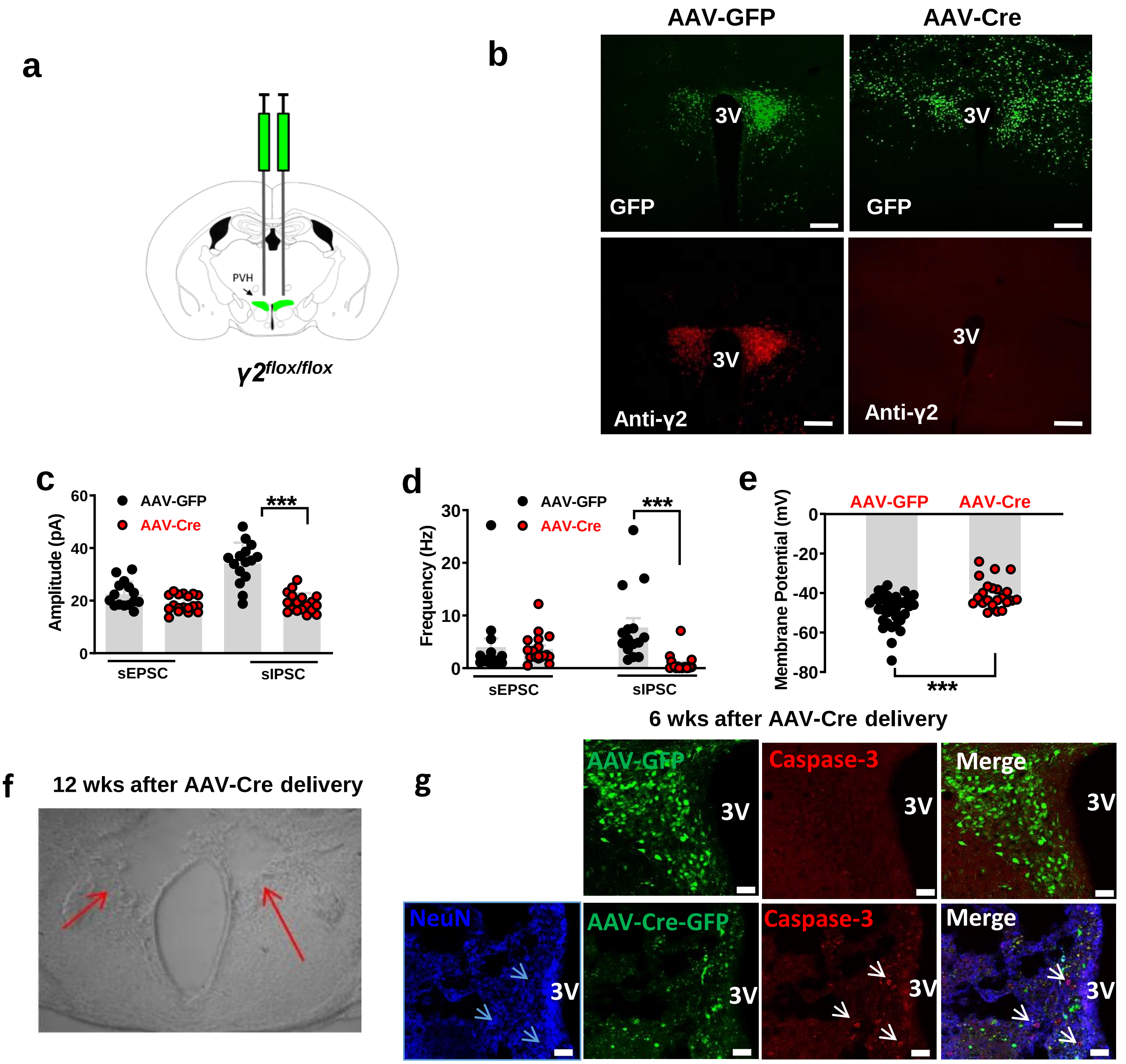

Supplementary Fig. 6. Bilateral deletion of GABA-A γ2 subunit disrupts GABAergic inputs to the PVH and caused neuron death. (a) Schematic diagram showing delivery and expression of AAV-GFP or AAV-Cre-GFP to PVH of $\gamma 2^{flox/flox}$ adult mice (10-12wks). (b) GFP and immunostaining for γ2 (red) showing expression of AAV-GFP and AAV-Cre-GFP bilateral delivered (top panels) and immunostaining of γ2 subunit in representative PVH sections (bottom panels) of mice 6wks after virus delivery. (c-d) Quantification of the amplitude (c, (unpaired 2-tailed Student's t test, n=15 for GFP or 18 for Cre, t=3.665, df=54, ***p=0.0006)) and frequency (d, unpaired Student's t test, n=13 for GFP or 14 for Cre, t=3.665, df=54, ***p=0.0006)) of sIPSCs and sEPSCs recorded from PVH neurons in mice 3wks after AAV-GFP and AAV-Cre-GFP delivery. (e) Membrane potential (mV) from mice 3wks after AAV-GFP or AAV-Cre-GFP delivery (unpaired 2-tailed Student's t test, n=34 for GFP or 22 for Cre, t=3.665, df=54, ***p=0.0006). (f) A representative picture showing morphology of PVH 12 weeks after AAV-Cre-GFP delivery to $\gamma 2^{flox/flox}$ adult mice. (g) Pictures showing AAV-GFP (top panels) and AAV-Cre-GFP (bottom) with immunostaining of cleaved Caspase 3 (red), demonstrating significant more expression of cleaved Caspase 3 expression in AAV-Cre-GFP injected mice (while arrows). The panel with blue NeuN immunostaining showing tissue morphology. Data are represented as mean ± SEM. Scale bars: 100μm. AAV: adeno associated viral vectors.

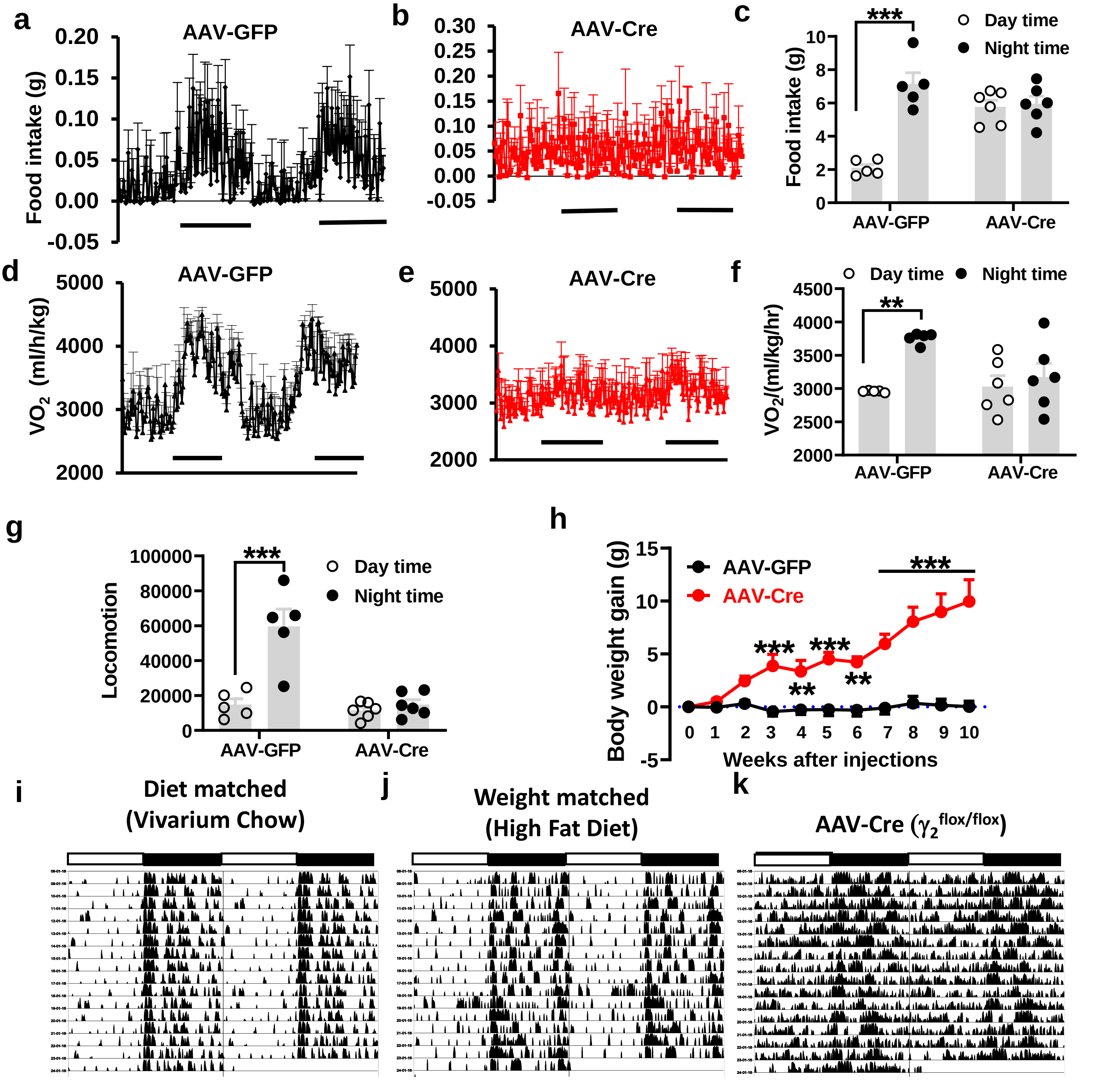

Supplementary Fig. 7. Bilateral deletion of GABA-A γ2 subunit in PVH led to loss of diurnal feeding, O_2 consumption and locomotion activity patterns, and resulted in obesity. (a-f) Adult γ2$^{flox/flox}$ mice (10-12wks old males) received AAV-GFP or AAV-Cre-GFP in bilateral PVH. Food intake (a, b and c, 2-way ANOVA, n=5 for GFP and 6 for Cre, F(6,84)=3.382, df=6, ***p<0.0001, GFP day vs night; p=0.9914, Cre day vs night), O_2 consumption (d, e and f, 2-way ANOVA, n=5 for GFP and 6 for Cre, F(1, 18)=5.002, df=6, **p<0.0081, GFP day vs night; p=0.8816, Cre day vs night) and locomotion (beam breaks, 2-way ANOVA, n=5 for GFP and 6 for Cre, F(6,117)=7.258, df=6, ***p<0.0001, GFP day vs night; p=0.9601, Cre day vs night) were measured using CLAMS 6-8wks after the viral injections. Data were averaged for 48h. (h) Weekly body weight (2-way ANOVA, n=11 for GFP and 8 for Cre, F(10, 165)=8.159, df=10, ***p<0.0001 for body weight at10 weeks after viral injection) changes were monitored for 10 wks after delivery with the viral vectors. Data are represented as mean ± SEM. (i-k) Voluntary activity measured by running wheels in home age in control (i), body weight matched group (j) and the γ2 deletion group (k).

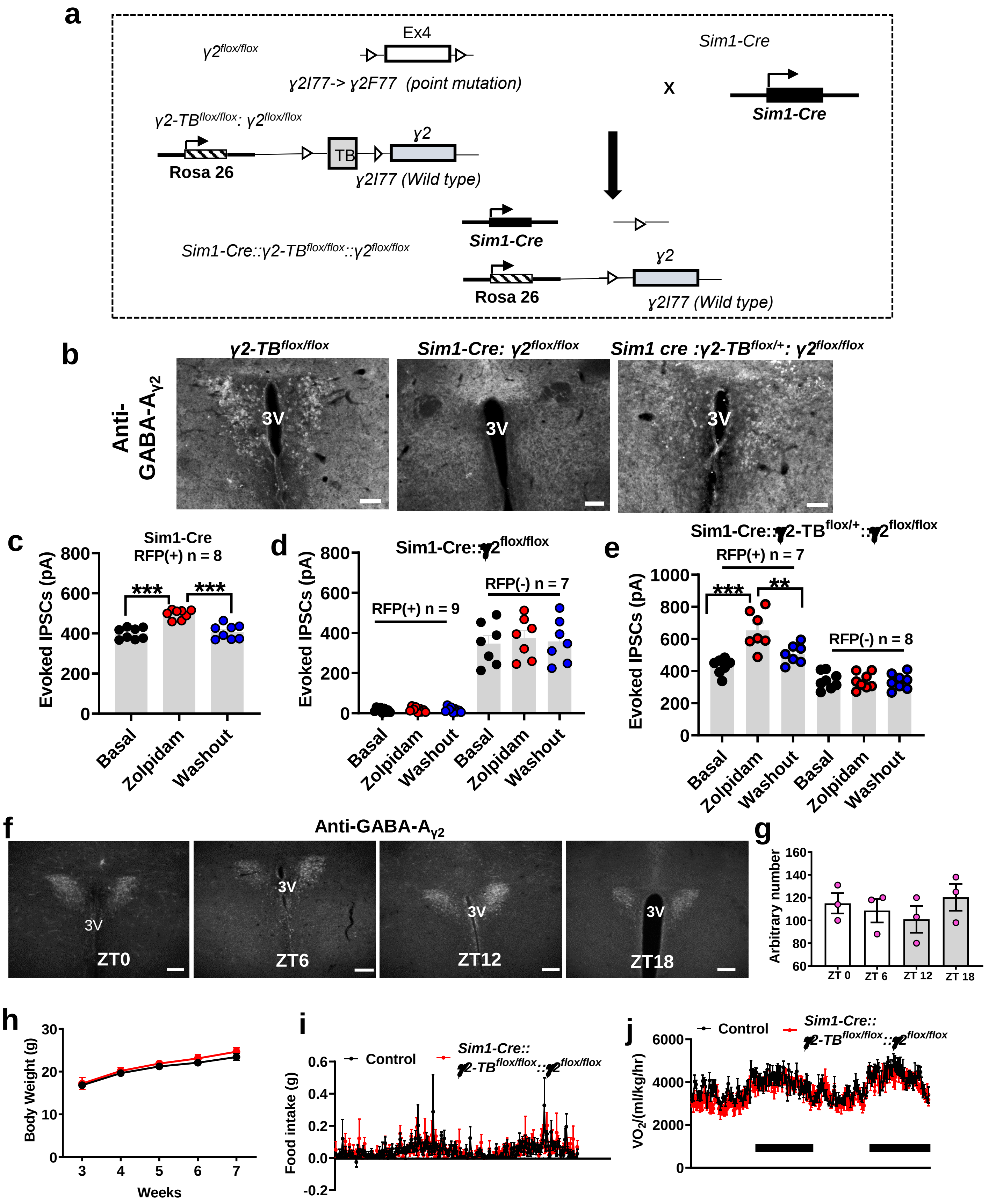

Supplementary Fig. 8: legend in the next page.

Supplementary Figure 8. Generation and verification of mice with GABA-A γ2 subunit expression clamped at a constant level in Sim1 neurons of PVH. (a) Schematic illustration showing generation of mice. (b) Representative images of immunostaining for γ2 subunit replacement. Expression of γ2 was normal in controls (left), deletion in the PVH (middle) and re-expression (right) of the indicated genotypes. (c-e) Evoked IPSCs in PVH neurons were performed on Sim1-Cre neurons in PVH identified by tdTomato (RFP) expression (breeding with reporter Ai9 mice) in brain sections of *Sim1-Cre* (c, 1-way ANOVA, n=8/each, F(6,84)=3.382, df=6, ***p<0.0001, GFP day vs night; p=0.9914, Cre day vs night), *Sim1-Cre:: γ2*$^{flox/\mathrm{flox}}$ (d, 1-way ANOVA, n=9 for GFP and 7 for Cre, F(6,84)=3.382, df=6, p<0.0001, GFP day vs night; p=0.9914, Cre day vs night) and *Sim1-Cre:: γ2*$^{flox/flox}$: *γ2-TB*$^{flox+}$ mice (e, 1-way ANOVA, n=7 for GFP and 8 for Cre, F(6,84)=3.382, df=6, ***p<0.0001, GFP day vs night; p=0.9914, Cre day vs night). RFP negative neurons were used as control of non-Cre mediated events. Recording were processed with saline (basal), Zolpidem treatment (Zol) and washout conditions. PVH neurons in control mice showed normal potentiating effects on eIPSCs by Zol. In Sim1-Cre:: *γ2*$^{flox/flox}$ mice, PVH Sim1 neurons exhibited dramatic reduction in eIPSCs and non-Sim1 neurons showing no potentiating effects of eIPSCs by Zol, consisting with γ2 deletion in Sim1 neurons and the point-mutation (I77→F77) in γ2$^{flox/flox}$ alleles. In Sim1-Cre:: *γ2*$^{flox/\mathrm{flox}}$: *γ2-TB*$^{flox+}$ mice, Sim1 neurons showed potentiating effects of eIPSCs by Zol while non-Sim1 neurons showed no potentiating effects, consistent with a successful reexpression of wild type γ2 subunit. Data are represented as mean ± SEM. Scale bars: 100μm. 3v: third ventricle. (f-h) Immunostaining (f) and quantification of γ2 subunit expression at ZT0, 6, 12 and 18 in *Sim1-Cre:: γ2*$^{flox/flox}$: *γ2-TB*$^{loxp/\mathrm{flox}}$ mice (g, n=3 each), weekly body weight from 3wk to 7wks of age in control and *Sim1-Cre:: γ2*$^{flox/flox}$: *γ2-TB*$^{flox/flox}$ mice (n=5 for Control and 6 for γ2 mice); real time traces showing feeding pattern for 48h (i) and (j) O_2 consumption for 48h in mice of control and *Sim1-Cre:: γ2*$^{flox/flox}$: *γ2-TB*$^{flox/flox}$ (n=5-6 males/group).

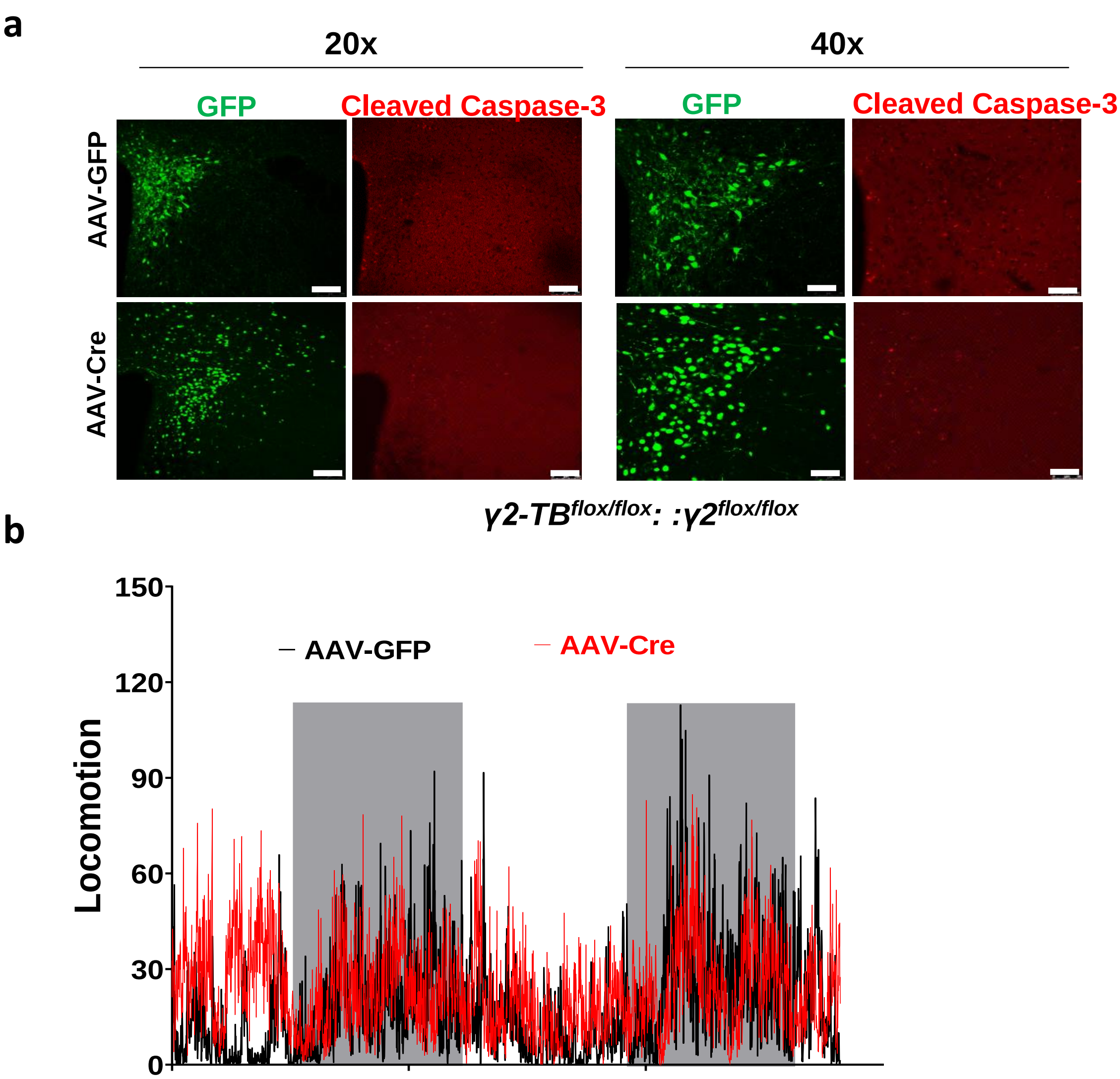

Supplementary Fig. 9. Assessment of apoptosis in PVH neurons and locomotion of *γ2-TB*$^{flox/flox}$ ::*γ2*$^{flox/flox}$ mice with delivery of AAV-Cre-GFP to PVH to clamp γ2 expression at a constant level. (a) Evaluation of neuronal cell death by immunostaining with anti-cleaved caspase-3 (Red) in PVH of mice 14-15 wks after viral vector delivery (GFP). Right panels representing 2X amplification of pictures shown in the left panels for a better view of caspase 3 expression. The expression of cleaved-caspase was comparable between the groups. (b) Locomotor activity measured by beam break in CLAMS 6-7 weeks after viral delivery to bilateral PVH of of *γ2*$^{flox/flox}$::*γ2-TB*$^{flox/flox}$ mice. Scale bar: 100uM.
